# Duplicate gallbladder: A case report of a patient with cholecystitis after cholecystectomy^☆^

**DOI:** 10.1016/j.ijscr.2019.10.075

**Published:** 2019-11-03

**Authors:** Samuel J. Pera, Noah Huh, Sonia T. Orcutt

**Affiliations:** Department of Surgery, University of Illinois College of Medicine at Peoria, 624 NE Glen Oak Avenue, Peoria, IL 61603, USA

**Keywords:** Duplicate gallbladder, Cholecystitis, Magnetic resonance cholangiopancreatography, Case report

## Abstract

**Introduction:**

Duplicate gallbladder is a congenital anomaly with various anatomical presentations that can pose difficult diagnostic dilemmas. This case presents the consequence of recurrent cholecystitis after prior cholecystectomy due to delay in diagnosis of a duplicate gallbladder and insufficient treatment at first presentation. It also provides the opportunity to discuss the anatomical variations of duplicate gallbladders and their clinical implications.

**Presentation of case:**

We report on a 46-year-old woman who presented with symptoms of cholecystitis despite a history of cholecystectomy. Magnetic resonance cholangiopancreatography (MRCP) as well as review of intraoperative cholangiogram from the index surgery identified a cystic structure continuous with the biliary tree. Laparoscopic cholecystectomy was performed and histology confirmed a duplicate gallbladder. The patient did well post-operatively without any complications.

**Discussion:**

Harlaftis’s classification of duplicate gallbladder categorizes anatomical variations based on embryological origin. Though rarity contributes to missed diagnosis, modern imaging techniques that delineate the biliary tree can identify these abnormalities. Recognizing these variations can identify risk for recurrent disease preoperatively and thereby guide surgical decision-making.

**Conclusion:**

Duplicate gallbladder poses a risk for the unique presentation of recurrent cholecystitis despite cholecystectomy. Advanced imaging techniques that demonstrate biliary anatomy can identify duplicate gallbladder perioperatively. For those presenting with disease in any one gallbladder, resection of both is ideal to prevent recurrence of disease.

## Highlights

• Cholecystitis after cholecystectomy raises suspicions for duplicate gallbladder.
• Imaging techniques demonstrating biliary anatomy can diagnose duplicate gallbladder.
• Duplicate gallbladders arise from either a common or duplicate primordium.
• Gallbladder disease is sufficient indication for resection of duplicate gallbladder.

## 1 Introduction

Duplication of the gallbladder is a rare congenital anomaly that spontaneously occurs due to embryological defects in the 5th and 6th pharyngeal pouches, with an estimated incidence of 1 in 4000 births [1]. Given its rarity and often functionally silent nature, a duplicate gallbladder is typically not in the differential diagnosis for abdominal pain after prior cholecystectomy. Even when it may be considered, it can be challenging to detect, as preoperative imaging can miss up to 50% of cases [[2], [3], [4]]. Here we discuss a case referred to a tertiary referral center where despite anomalous findings on perioperative imaging, the duplicate gallbladder was missed and ultimately lead to recurrence of cholecystitis after the initial cholecystectomy. This work has been reported in line with the SCARE criteria [5], and the patient provided informed consent.

## 2 Presentation of case

A 45-year-old woman referred to our tertiary referral center presented with a 1 month history of right upper quadrant pain exacerbated by meals with associated nausea and occasional abdominal distention. She had a surgical history of laparoscopic cholecystectomy 3 years prior and a Roux-en-Y gastric bypass with subsequent re-exploration due to internal hernia, both 1 year prior. She reported her symptoms resembled the pain she felt prior to her cholecystectomy.

Abdominal examination demonstrated tenderness to palpation in the right upper and lower quadrants; no organomegaly or hernia defects were detected. Liver function tests identified marginally elevated AST (35 u/L) and alkaline phosphatase (154 u/L). CT scan done by her surgeon for evaluation showed a fluid collection in the gallbladder fossa of unclear etiology. Due to the persistent symptoms, she was referred for a second opinion to our tertiary care center.

**Fig. 1:**
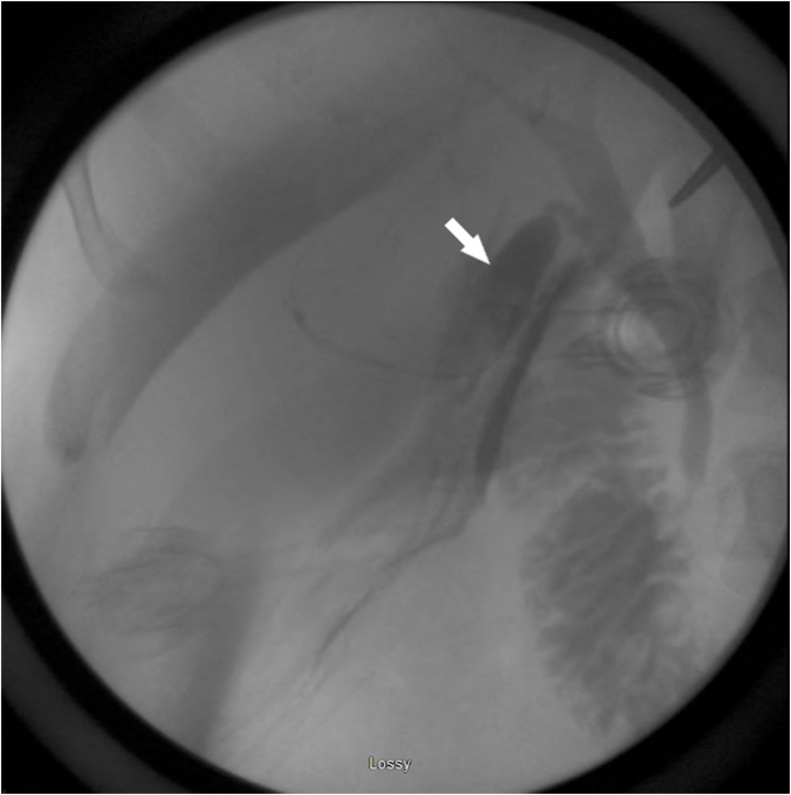
Intraoperative cholangiogram during index cholecystectomy. Contrast demonstrates a normal common bile duct and intrahepatic ducts. An additional cystic structure (arrow) contiguous with the common bile duct can be seen.

**Fig. 2:**
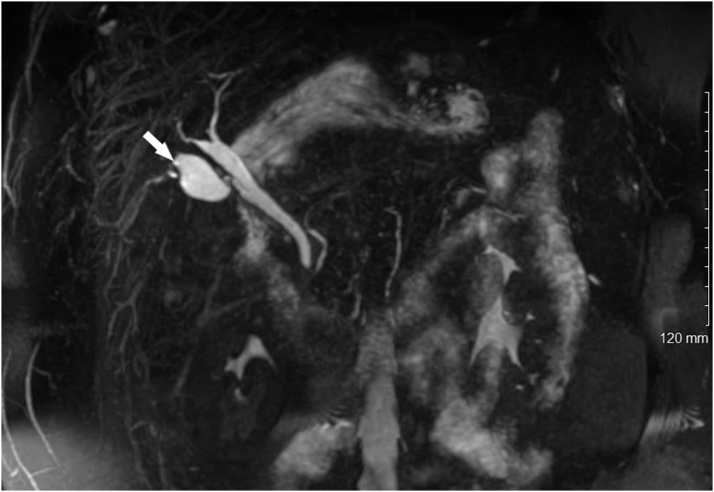
MRCP performed before the second cholecystectomy. A gallbladder-like structure in the gallbladder fossa demonstrates flow through an apparent cystic duct. Signal void from the prior cholecystectomy clips can be seen at the superolateral aspect of the duplicate gallbladder (arrow).

The differential diagnosis at that time included a remnant infundibulum from subtotal cholecystectomy, a pseudocyst of the common bile duct, a choledochal cyst, a recurrent internal hernia with postoperative changes on imaging, and a duplicate gallbladder. Review of her prior records showed that there was no aberrant anatomy noted during her initial operation, and that a cholangiogram was completed with no clear abnormalities identified by the surgeon at the time (Fig. 1). Pathology confirmed a gallbladder with a cystic duct and chronic cholecystitis. Magnetic resonance cholangiopancreatography (MRCP) was therefore obtained to clarify the diagnosis. This demonstrated the prior surgical clips, as well as a cystic structure in the gallbladder fossa contiguous with a normal-appearing common bile duct through an apparent cystic duct (Fig. 2). Of note, the appearance of this structure was thought to be identical to a native gallbladder, were it not for the history of previous cholecystectomy. Therefore, she was offered surgery for presumed duplicate gallbladder.

**Fig. 3:**
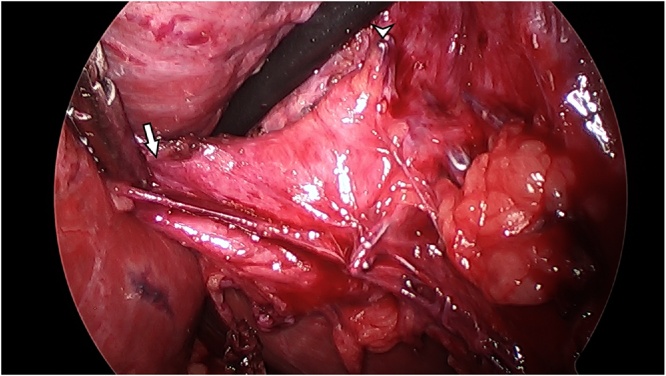
Intraoperative laparoscopic image from the second cholecystectomy. The duplicate gallbladder is splayed laterally (arrow). Surgical clips from the prior cholecystectomy are noted in the right side of the image (arrowhead). Dissection confirmed a duplicate gallbladder complete with its own cystic artery and duct.

**Fig. 4:**
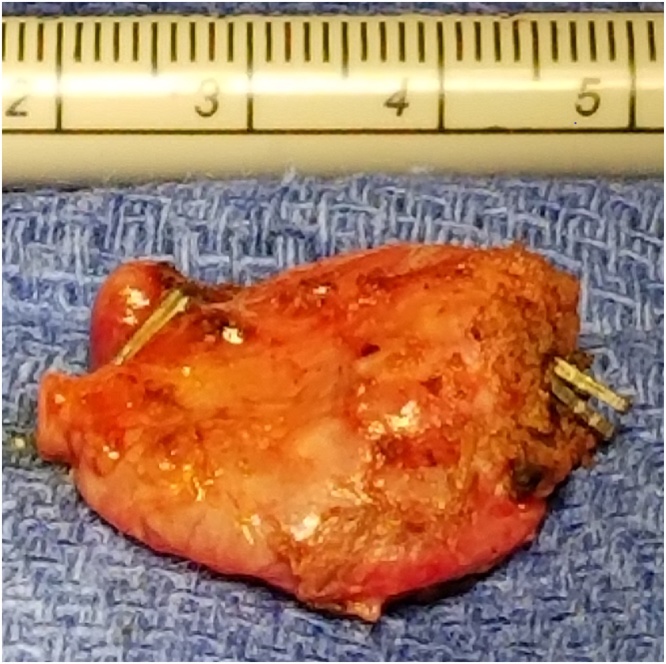
Duplicate gallbladder post-resection. The duplicate gallbladder was resected in its entirety intact with the surgical clips denoting the origin of the duplicated cystic artery and duct.

She was taken to the operating room and successfully underwent laparoscopic cholecystectomy. On dissection of the prior gallbladder fossa, clips from the prior cholecystectomy were identified along with the anomalous biliary structure inferior and lateral to the noted clips (Fig. 3), which appeared to be a diminutive gallbladder with an isolated cystic duct and artery. The gallbladder was situated directly above the right hepatic artery, from which arose the cystic artery supplying the duplicate gallbladder. Intraoperative cholangiogram through the cystic duct demonstrated drainage into the common bile duct and bifurcating hepatic ducts, with no additional structures. The duplicate gallbladder was resected without complication after which external and internal examination demonstrated an intact organ with a duct (Fig. 4). Pathology confirmed gallbladder tissue with mild, chronic inflammation. The patient had an unremarkable postoperative course. Her symptoms subsequently resolved, and 2 years later she remains asymptomatic.

## 3 Discussion

Duplicate gallbladders can be challenging to diagnose since they are rare congenital anomalies and because of a wide variation of presentations secondary to the multitude of embryologic etiologies. The variations were first classified by how the gallbladders drained into the common bile duct. Vesica fellea divisa denoted duplicate gallbladders sharing a common cystic duct while vesica fellea duplex described gallbladders draining through separate cystic ducts [1].

The modern classification by Harlaftis takes embryological origin into consideration. Type 1 duplicate gallbladders arise from the same primordium but split later in development. These Type 1 gallbladders can often be identified by a common cystic duct that drains both gallbladders. The septate gallbladder is a single cystic structure separated into two by an involuting wall, though both gallbladders are joined at the base where they drain into a single cystic duct. In V-shaped variants the two gallbladders each have cystic ducts, which drain at a shared point along the common bile duct. In Y-shaped variants each gallbladder drains into its own cystic ducts, but these cystic ducts join to form a common cystic duct that drains into the common bile duct [6].

In Type 2 variants, a distinctly separate primordia gives rise to the duplicate gallbladder which has its own second cystic duct and is often positioned more distantly than the Type 1 variants. This accessory gallbladder group is further subcategorized by the accessory gallbladder’s position in the biliary tree; ductular types drain into the common bile duct whereas trabecular types drain into either the right or left hepatic ducts [6]. In our patient’s case the duplicate gallbladder had a separate cystic duct draining into the common bile duct, thereby representing a Type 2 ductular gallbladder.

Appropriate diagnosis is important to avoid recurrence of disease, repeat procedures, and especially surgical complications secondary to distorted biliary anatomy [7]. Ultrasonography is the most popular imaging modality for assessing gallbladder disease. However, its primary utility comes from assessing the gallbladder wall and the gallbladder’s contents, not the anatomy of the biliary tree. Thus, the differential diagnosis becomes highly nonspecific including not only for duplicate gallbladder but also the following: Phrygian cap gallbladder, gallbladder diverticulum, choledochal cyst, folded gallbladder, focal adenomatosis, and, in post-surgical cases, remnant gallbladder tissue [1,8,9]. The variations of duplicate gallbladder, especially Type 2 variants, can be more difficult to detect when the duplicate organ is positioned remotely or deeper in the viscera, e.g. above the right or, though rarely, the left hepatic artery [1,2,8].

Perioperative imaging options include abdominal CT scans, hepatobiliary iminodiacetic scanning, endoscopic retrograde cholangiopancreatography, transcutaneous cholangiography, or intraoperative cholangiograms, all of which delineate the biliary tree to varying precision [[8], [9], [10], [11], [12]]. However, magnetic resonance cholangiopancreatography (MRCP) and ERCP more precisely illustrate complicated anatomy even in disease states [[8], [9], [10], [11], [12]]. MRCP has the added advantage of not only imaging the biliary tree but also the viscera itself to aid in diagnosis.

In reference to the extent of surgical therapy for patients with duplicate gallbladder, clinicians should be aware that duplicate gallbladders pose a unique risk of recurrent biliary complications despite cholecystectomy. The occurrence of a duplicate gallbladder alone does not increase the risk of infectious biliary disease in an individual and thus does not warrant further investigation if noted incidentally [8]. Each gallbladder has an equal risk for disease, but the disease states appear to be independent, i.e. cholecystitis in one gallbladder does not cause cholecystitis in the other [2,13]. However, in patients with duplicate gallbladder and infectious biliary disease, the patient has demonstrated risk factors for the development of infectious biliary disease in both gallbladders. Therefore, resection of both gallbladders is recommended to avoid the chance of recurrence and the need for another abdominal surgery [2,8,13].

## 4 Conclusion

Duplicate gallbladder poses a risk for the unique presentation of recurrent cholecystitis despite cholecystectomy. For those presenting with disease in any one gallbladder, resection of both is ideal to prevent recurrence of disease.

## Declaration of Competing Interest

We the authors Samuel J. Pera, Sonia T. Orcutt, and Noah Huh do not have conflicts of interest to declare.

## Funding

This research did not receive any specific grant from funding agencies in the public, commercial, or not-for-profit sectors.

## Ethical approval

Our institution does not require IRB or ethics approval for case reports.

## Consent

Written informed consent was obtained from the patient for publication of this case report and accompanying images. A copy of the written consent is available for review by the Editor-in-Chief of this journal on request.

## Author contribution

**Sonia T. Orcutt:** Conceptualization, Methodology, Validation, Formal Analysis, Investigation, Resources, Writing – Original Draft, Writing – Review & Editing, Visualization, Supervision, Project Administration. **Samuel J. Pera:** Conceptualization, Methodology, Validation, Formal Analysis, Investigation, Resources, Writing – Original Draft, Writing – Review & Editing, Visualization, Supervision, Project Administration. **Noah Huh:** Formal Analysis, Investigation, Writing – Original Draft, Writing – Review & Editing, Visualization.

## Registration of research studies

This is not a “first in man” study, requiring registration as per the journal Guide for Authors.

## Guarantor

As authors of the study, Samuel J. Pera, Sonia T. Orcutt, and Noah Huh agree to be the Guarantors of this case study.

## Provenance and peer review

Not commissioned, externally peer-reviewed.
